# Maternal obesity increases the risk of metabolic disease and impacts renal health in offspring

**DOI:** 10.1042/BSR20180050

**Published:** 2018-03-29

**Authors:** Sarah J. Glastras, Hui Chen, Carol A. Pollock, Sonia Saad

**Affiliations:** 1 Department of Medicine, Kolling Institute, University of Sydney, Sydney, Australia; 2 Department of Diabetes, Endocrinology and Metabolism, Royal North Shore Hospital, St Leonards, NSW 2065, Australia; 3 School of Life Sciences, Faculty of Science, University of Technology Sydney, Australia

**Keywords:** chronic kidney disease, developmental programming, epigenetics, fetal programming, maternal obesity, metabolic

## Abstract

Obesity, together with insulin resistance, promotes multiple metabolic abnormalities and is strongly associated with an increased risk of chronic disease including type 2 diabetes (T2D), hypertension, cardiovascular disease, non-alcoholic fatty liver disease (NAFLD) and chronic kidney disease (CKD). The incidence of obesity continues to rise in astronomical proportions throughout the world and affects all the different stages of the lifespan. Importantly, the proportion of women of reproductive age who are overweight or obese is increasing at an alarming rate and has potential ramifications for offspring health and disease risk. Evidence suggests a strong link between the intrauterine environment and disease programming. The current review will describe the importance of the intrauterine environment in the development of metabolic disease, including kidney disease. It will detail the known mechanisms of fetal programming, including the role of epigenetic modulation. The evidence for the role of maternal obesity in the developmental programming of CKD is derived mostly from our rodent models which will be described. The clinical implication of such findings will also be discussed.

## Introduction

Obesity affects almost one quarter of the adult population and is increasing rapidly amongst young women globally, with 30–50% of women of childbearing age falling within the spectrum of being overweight to obese [1–3]. The significant increase in maternal obesity over the last decade has had ramifications for all aspects of female reproductive health, with maternal adiposity strongly associated with an increased risk of almost all maternal and fetal complications. Although genetic predisposition and postnatal environment are key features for the development of chronic disease, there appears to be a critical window during gestation which influences the long-term risk unaccounted for by genetic tendency and postnatal environment alone.

Maternal obesity has lasting effects on the long-term health of offspring. Evidence from both human and animal studies suggests that maternal obesity ‘programs’ the offspring toward obesity, hyperglycemia, diabetes, and hypertension, all key features of the metabolic syndrome [4,5]. This observation evokes the concept of the developmental origins of health and disease; a concept first explored by Barker and Martyn, which suggests that chronic disease may be influenced by *in utero* exposure to the maternal milieu [6]. There is substantial evidence that maternal obesity increases risk of diabetes, obesity, hypertension, cardiovascular disease, and even premature death in adult offspring [7–10]. The effect of maternal obesity on the risk of chronic kidney disease (CKD) in offspring is much less understood.

The current review will discuss the evidence for the role of maternal obesity in the developmental programming of chronic metabolic disease in offspring, particularly focussing on the role of fetal programming in the development of CKD. The mechanisms of fetal programming, as it may relate to CKD, will be reviewed highlighting the role of inflammation, oxidative stress, and epigenetic changes. The avenues for future research will be discussed.

## Maternal obesity programs metabolic disease in offspring

When an unborn fetus is exposed to the intrauterine environment associated with maternal obesity, it has lasting effects on the offspring’s long-term metabolic health, independent of genetic predisposition and postnatal environmental factors [7,8,11–17]. Both human observational studies and animal models of maternal obesity have contributed to our understanding of the role of fetal programming in chronic disease risk. Specifically, epidemiological studies have shown that offspring born to obese mothers are at an increased risk of obesity, type 2 diabetes (T2D), cardiovascular disease, and non-alcoholic fatty liver disease (NAFLD) (Table 1). A large, retrospective cohort study of over 100,000 person years found that offspring of obese mothers (body mass index (BMI) >30) had a 35% increased mortality, mostly due to cardiovascular death, compared to offspring of normal weight mothers [7]. Using a similar experimental design, Eriksson et al. [14] also found that higher maternal BMI was strongly associated with an increased risk of cardiovascular and cerebrovascular diseases, both coronary heart disease and stroke. There are currently no observational studies that specifically examine the effect of maternal obesity on offspring’s long-term kidney health.

**Table 1:** Summary of epidemiological studies assessing the effect of maternal obesity on offspring risk of chronic disease

Study name	Year of publishing	Country	Study design	Sample size	Outcome of interest	Main findings	Adjusted variables	*P-*value	Level of evidence
**Obesity in adulthood**									
**Eriksson et al**. [11]	2015	Denmark	Retrospective cohort	2003	Adult BMI (mean age: 62 years)	Higher maternal BMI was associated with significantly higher BMI in offspring	Current age	*P*=0.002	III-3
**Schack-Nielsen et al**. [12]	2010	Denmark	Retrospective cohort	1540	Adult BMI (mean age: 42 years)	Higher gestational weight gain was associated with significantly higher BMI in offspring	Sex, maternal age/pre-pregnancy, BMI, parental social status, education/single-mother status, prematurity, birth weight, and smoking	*P*=0.003	III-2
**Laitinen et al**. [13]	2001	Finland	Retrospective cohort	6280	Adult BMI (mean age: 31 years)	Offspring overweight/obesity was more common if the mother was overweight/obese during pregnancy, BMI at the age of 31 correlated with BMI at the age of 14	N/a	*P*<0.001	III-3
**Cardiovascular disease in adulthood**									
**Eriksson et al.** [14]	2014	Finland	Retrospective hospital archive medication register	13345	Cardiovascular disease (coronary heart disease and stroke)	Higher maternal BMI was associated with increased risk of cardiovascular disease; as well as coronary heart disease and stroke in offspring when analyzed separately (*P*=0.003 and *P*=0.04, respectively)	Childhood socioeconomic status, adult socioeconomic status, income, education, sex, and year of birth	*P*=0.002	
**Reynolds et al**. [7]	2013	Scotland	Retrospective cohort	37709	All-cause mortality	Offspring of obese mothers had a 35% higher risk of mortality compared with offspring of mothers of normal weight. Offspring of obese mothers had an increased risk of cardiac-related hospitalization	Maternal age at delivery, socioeconomic status, offspring sex, birth weight, gestation at delivery, and gestation at measurement of BMI	HR: 1.17–1.55	III-2
					Hospitalized for a cardiovascular event				
**Forsen et al**. [8]	1997	Denmark	Retrospective cohort	3300 (men only)	Death from coronary heart disease (ICD)	Higher maternal BMI was associated with increased risk of death from cardiovascular disease	N/a	*P*=0.008	III-3
**T2D in adulthood**									
**Eriksson et al.** [14]	2014	Finland	Retrospective hospital archive/medication register	13345	T2D (as determined by use of antidiabetic medications)	The risk of T2D was increased with higher maternal BMI; the association was stronger in women	Childhood and adult socioeconomic status, income, education, sex, and year of birth	*P*=0.004	III-3
**NAFLD**									
**Patel et al**. [15]	2016	U.K.	Prospective pregnancy cohort	1581	NAFLD as determined by liver ultrasound at the age of 17–18 years	Maternal overweight/obesity and pre-pregnancy BMI were associated with greater odds of NAFLD in offspring, even when adjusting for confounders (lost significance when adjusted for neonatal offspring adiposity)	Age at assessment, gender, maternal age at delivery, parity, maternal pre-pregnancy alcohol intake, household social class, birth weight	*P*<0.05	III-2

This table highlights major studies that have identified the significant influence of maternal obesity on chronic disease risk. Level of evidence is derived from NHMRC levels of evidence where level I evidence is a systematic review of level II evidence, II is a randomized controlled trial, III is a comparative study; -2 concurrent controls, -3 historical controls or two single arm studies [18].

The aforementioned studies mostly employ observational, epidemiological designs, inherently subject to differences in selection methods, measurement of study variables, design-specific sources of bias, control of confounding variables and statistical analyses [19]. For example, many studies have used self-reported pre-pregnancy BMI rather than directly measuring BMI, and some studies did not have additional measurements of body weight during pregnancy. Breastfeeding is infrequently identified as a confounding factor though it is known to be less common in obese women and is well known to protect offspring against risk of adult obesity and T2D [20–24]. Animal studies allow controlled experimental design and can induce a specific maternal perturbation to determine its influence on offspring’s long-term health. Many animal studies have utilized dietary manipulation in rodent models to examine the effects of maternal obesity on the offspring. Table 2 summarizes some examples of long-term studies using various species to model maternal obesity. These studies have overwhelmingly supported the concept that maternal obesity programs the development of metabolic disease in adult and even aged offspring, and that maternal obesity compounds the effect of diet-induced obesity in adulthood. Rodents, rabbits, guinea pigs, sheep, and non-human primates have been used to model maternal obesity and determine its effects on offspring’s health. The largest meta-analysis including 53 rodent studies confirmed that maternal obesity is associated with significantly higher body weight in offspring post weaning [25].

**Table 2:** Metabolic sequelae of maternal obesity in animal models

Study	Year	Species/strain	Maternal diet (fat %)	Comparator group	Age of offspring	Main findings
**Ramirez-Lopez et al.** [26]	2015	Wistar rat	Cafeteria (*ad libitum*)	Offspring of chow-fed mothers	Week 20	Male offspring from obese mothers showed significantly greater abdominal fat than control offspring although no significant difference in body weight between the groups was found.
**Srinivasan et al**. [27]	2006	SD rat	HFD (60%)	Offspring of chow-fed mothers	Day 60	Offspring of HFD-fed mothers had increased glucose, free fatty acids, triglycerides, and glucose intolerance.
**Rajia et al.** [28]	2010	SD rat	HFD (60%)	Offspring of chow-fed mothers	Week 21	HFD-fed offspring of HFD-fed mothers had increased body weight, fat mass, and glucose intolerance with increased insulin, leptin, insulin resistance, and hyperphagia compared with offspring of chow-fed mothers.
**Chen et al.** [29]	2014	SD rat	HFD (60%)	Offspring of chow-fed mothers, offspring fed chow compared with HFD	Week 9	Offspring of HFD-fed mothers had increased adiposity, hyperinsulinemia, hyperlipidemia, and insulin resistance.
						Only offspring of HFD-fed mothers who were fed HFD had impaired glucose tolerance, and not those fed chow.
**Buckley et al.** [30]	2005	Rat	HFD (59%)	Offspring of chow-fed mothers	3 months	Offspring of HFD-fed mothers had increased proportions of both total body fat and abdominal fat, hyperinsulinemia on oral glucose tolerance test at 15 min and elevated liver triglyceride content. Insulin signaling protein expression levels in the offspring of HFD-fed mothers were consistent with reduced hepatic insulin sensitivity.
**Bayol et al.** [31]	2007	Rat	Cafeteria	Offspring of chow-fed mothers	Week 10	Offspring of junk food-fed mothers exhibited increased body weight and BMI compared with all other offspring
**Blackmore et al.** [32]	2014	SD rat	High fat and sugar (20%)	Offspring of chow-fed mothers	Week 12	Although offspring of high fat/high sugar mothers had the same body weight and adiposity as offspring of chow-fed mothers, their heart mass was greater, ventricular volumes were increased, and there was increased ventricular wall thickening.
**Samuelsson et al.** [33]	2008	C57BL/6 mouse	Obesogenic diet (16% fat/33% sugar)	Offspring of chow-fed mothers	3 and 6 months of age	At 6 months, offspring of obese mothers were heavier, with increased adiposity, endothelial dysfunction, hypertensive, and significantly reduced skeletal muscle mass. Fasting insulin was raised at 3 months and by 6 months fasting glucose was elevated
**King et al.** [34]	2014	C57BL/6	Cafeteria (58% fat/25% sugar)	Offspring of chow-fed mothers	3 and 6 months of age	At 3 months: post-weaning exposure to cafeteria diet increased glucose, insulin, and cholesterol in males; increased plasma insulin and cholesterol in females and increased HOMA-IR in both sexes. There was an additive effect of maternal overnutrition to increase insulin levels in males.
						At 6 months: no additional effect of maternal overnutrition was seen.
**Castaneda-Gutierrez et al.** [35]	2011	Guinea pig	HFD (40%)	Offspring of chow-fed mothers	Day 136	Feeding a HFD during pregnancy induced a 3% increase in body fat in the neonates without change in birth weight. A maternal HFD increased the offspring’s adiposity at 2 and 21 days but had no effect on body composition later in life.
**Long et al.** [36]	2010	Sheep	150% nutrient requirements (obseogenic diet)	Offspring of chow-fed mothers	2 years old	Fasting glucose was greater; glucose effectiveness and insulin sensitivity were lower in offspring from obese compared with control ewes. During a feeding challenge, offspring from obese ewes consumed approximately 10% more food and tended to have greater weight gain. Their percentage of body fat was greater
**McCurdy et al.** [37]	2009	*Macaca fuscata*	HFD (32%)	Offspring who were switched to normal diet (15% fat) at age 5 years	Age: 15 years	Chronic maternal HFD consumption, independent of maternal obesity, or diabetes, significantly increased the risk of NAFLD in the developing fetus that persisted into the postnatal period.
**Rivera et al.** [38]	2015	*Macaca fuscata*	HFD (37%)	Offspring of normal diet (15% fat)	13 months	Maternal obesity (defined as >15.8% body fat) but not maternal HFD consumption alone was associated with increased body weight. Offspring from HFD-obese mothers overconsumed high-fat/sucrose relative to control offspring demonstrating a preference for palatable HFD food.

This table demonstrates that maternal obesity is implicated in metabolic dysfunction utilizing a variety of species/strains and different offspring ages. It does not intend to provide an exhaustive list of all studies completed on the topic. Abbreviation: HFD, high-fat diet. HOMA-IR, homeostasis model assessment of insulin resistance. SD, Sprague-Dawley

## The role of maternal obesity in CKD risk in offspring

A limitation of the existing data regarding the effects of maternal obesity on metabolic health is that few studies, both in humans and animal models, have specifically examined the effect of maternal obesity on renal health in offspring. Several human studies have shown that offspring born to diabetic mothers are at increased risk of hypertension, hyperfiltration, and CKD [39]. A longitudinal study of over 5000 births found that overweight and obesity in early infancy are associated with increased risk of CKD in adulthood [40]. A large population-based, case–control study with 1994 patients with childhood CKD (<21 years of age at diagnosis) confirmed that maternal overweight and obesity were associated with a significantly increased risk of CKD in these young children (24 and 26% increased risk, respectively compared with the controls) [41]. Low birth weight, gestational diabetes, and maternal overweight/obesity were significantly associated with obstructive uropathy in children [41]. These clinical studies are suggestive that maternal obesity can negatively impact renal development and increase risk of CKD in later life.

Utilizing rodent models of maternal obesity, research within our laboratory has established that maternal obesity is a significant risk factor for the future development of CKD in offspring. In rodent models of maternal obesity utilizing high-fat diet (HFD) feeding (20 kJ/g, 43% fat, 21% protein, 36% carbohydrate) during gestation and lactation, we have demonstrated that offspring of obese mothers have increased fat deposition, insulin resistance, and impaired glucose tolerance together with increased albuminuria and renal pathology [42–46]. The kidneys of offspring of obese compared with lean mothers examined at Day 20, Week 9 and through to Week 32 showed persistent evidence of inflammation, oxidative stress, and fibrosis. Interestingly, postnatal feeding of HFD in offspring augmented the deleterious renal effects of maternal obesity, confirming the negative impact of persistent high calorie diet. Moreover, offspring of obese mothers were more prone to renal damage after an additional insult, such as streptozotocin-induced diabetes, suggesting maternal obesity may be an initial insult in a ‘two-hit’ model of disease. However, diet-induced HFD-feeding in offspring remained a very powerful means of inducing weight gain, glucose intolerance, albuminuria, and renal damage, which overpowers the effect of maternal obesity by postnatal week 32 [46]. Our results highlight that intrauterine exposure to maternal obesity predispose offspring toward CKD and implicates fetal exposure to maternal obesity as a significant risk factor for CKD.

Our previous studies established association with respect to maternal obesity and renal effects in offspring, however were not able to demonstrate direct causality. The kidneys of rodent offspring demonstrated deleterious changes including increased markers of oxidative stress, inflammation, and lipid deposition. Changes in blood glucose regulation and blood pressure by maternal obesity or postnatal insults may also have contributed to the renal effects. Indeed, rodent offspring of obese mothers are known to be hypertensive with evidence of endothelial dysfunction [33]. There has been some suggestions that early life exposure to hyperleptinemia may contribute to establishing hypertension in the offspring of obese mothers [47].

## Developmental programming of kidney disease in offspring

The influential role of developmental programming in the susceptibility to CKD has received greater attention in the context of nutritional deprivation, tobacco smoking, and gestational diabetes mellitus (GDM), as compared with maternal obesity [48]. The kidney is a highly vascular and metabolically active organ, particularly susceptible to the impact of prenatal insults [49]. Infants born prematurely, prior to 36 weeks of gestation have reduced nephron number and kidney size. Low birth weight, defined as birth weight below 2500 g, represents the postnatal manifestation of fetal growth restriction. Considerable evidence exists for an association between low birth weight and CKD [50–59]. Indeed, a recent meta-analysis found that low birth weight confers approximately 70% increased risk (OR: 1.77) of developing CKD in adult life, compared with normal birth weight [59]. Factors known to increase the likelihood of low birth weight include maternal nutritional deprivation, maternal smoking, placental insufficiency, twin pregnancy, and preterm delivery [48,60–63]. Mothers who smoke tobacco during pregnancy can permanently damage their offspring’s kidney health as a result of prematurity and low birth weight [64,65]. This is due to the disruption in delivery of nutrients to the growing fetus that leads to intrauterine growth restriction, which has permanent deleterious effects on renal health in postnatal life [66]. Though often associated with fetal overgrowth, maternal obesity can also conversely be associated with increased risk of premature birth and low birth weight, with implications for renal development and CKD risk [67].

Epigenetic mechanisms are implicated in the development of CKD [68,69]. As compared with genome-wide association studies (GWAS), epigenome-wide association studies (EWAS) have been more fruitful in demonstrating influence of epigenetic changes on CKD risk [70]. The tubuli from patients with CKD demonstrated significant changes in DNA methylation, particularly at enhancers associated with the increased expression of key fibrotic genes [71]. Furthermore, accelerated loss of renal function in patients with known CKD is associated with DNA methylation of genes involved in inflammation, as well as fibrosis [72] The role of epigenetics is increasingly understood to underpin the relationship between maternal perturbations and renal health in adulthood [73,74]. Much of this work in epigenetics and fetal programming has investigated the effects of intrauterine growth restriction and maternal smoking. There are no studies that have investigated the effect of maternal obesity on epigenetic changes specifically related to development of CKD. This needs to be addressed in future studies.

## Mechanisms of fetal programming affecting offspring kidney health

The total number of nephrons within the kidney is important, as each nephron provides a critical filtering function within the kidney. In humans, nephron endowment, defined as the number of nephrons at the start of postnatal life, is an important determinant of adult kidney health [75]. Nephrogenesis is complete by 32–36 weeks in humans and thereafter no new nephrons are formed. The total number of nephrons is correlated with birth weight in humans [51].

In the context of premature delivery or intrauterine growth restriction, nephron endowment is reduced and thus the normal physiological demand becomes overwhelmed leading to hyperfiltration and subsequent glomerular hypertrophy which may ultimately lead to CKD and systemic and intraglomerular hypertension [49,75,76]. The importance of kidney size is evident in neonates with retarded kidney growth during the first 18 months of life, who have increased risk of CKD in adulthood [77]. The underdeveloped kidney in offspring from smoking mothers is at least partially a direct result of the inflammatory and vasoconstrictive actions of nicotine and results in lower nephron endowment in the offspring [78]. Within our laboratory, in a rodent model of maternal smoking maternal smoking has been found to impair mitochondrial function, increase renal levels of reactive oxygen species, and reduce antioxidant defense mechanisms in the kidneys of adult offspring. The appearance of mitochondrial defects preceded the onset of albuminuria at postnatal week 13. Thus, mitochondrial damage caused by maternal smoking may play an important role in development of CKD in adult life [65]. Indeed, maternal smoking is associated with epigenetic changes which have been implicated in the development of CKD as well as cancer, rheumatoid arthritis, and other immune-mediated diseases [79–81].

**Figure 1:**
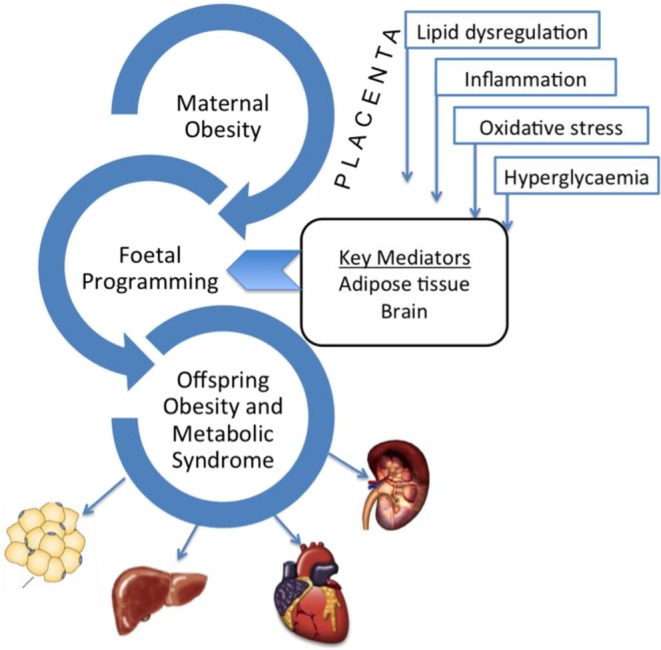
A schematic representation depicting the key players in developmental programming of maternal obesity

The mechanisms underpinning the association between maternal obesity and CKD are less well studied and have been pioneered by our research team. We confirm that inflammation, oxidative stress, and dyslipidemia are the key mechanisms involved in the relationship between maternal obesity and CKD [42–46]. The known mechanisms of developmental programming on chronic disease risk in offspring are depicted in Figure 1.

## Cross-talk amongst adipose tissue, placenta, and fetal kidney development in maternal obesity

### Adipose tissue has an important role in metabolic programming

Offspring of obese mothers are predisposed to adiposity, adipocyte hypertrophy, and weight gain in adulthood as a result of up-regulation of adipogenesis and lipogenesis [82]. Visceral adipose tissue is increased (including increased epididymal/periuterine, perirenal, omental, and mesenteric fat deposits), which has been shown to have particularly adverse metabolic consequences to the offspring in relation to insulin resistance and metabolic risk [83]. Key transcription factors involved in adipogenesis and lipogenesis include peroxisome proliferator-activated receptor-γ (PPARγ), CCAAT/enhancer binding protein, the sterol regulatory element-binding protein 1c as well as fatty acid synthesis enzymes such as fatty acid synthase. All of them are up-regulated in the adipose tissue of offspring of obese mothers [84]. PPARγ has been shown to be up-regulated in adipose tissue of offspring exposed to maternal obesity both prenatally and up to postnatal day 130 [85,86]. Fatty acid synthase and multiple fatty acid transporters have been shown to be up-regulated in retroperitoneal, omental, mesenteric, and subcutaneous fat deposits. Lipid accumulation, including cholesterol and phospholipid accumulation, within the glomeruli and proximal tubules is known to be associated with CKD [87]. We demonstrated that maternal obesity was associated with reduced renal function and increased fibrosis as measured by renal structural changes and fibronectin; renal inflammation and oxidative stress were up-regulated in the offspring of obese mothers. Table 3 shows the maternal anthropometric characteristics in mothers at the time of weaning, clearly demonstrating that HFD feeding in the mothers induced significant adiposity but not diabetes nor relative hyperglycemia in the offspring.

**Table 3:** Maternal anthropometric characteristics of dams at the time of weaning (day 21 postpartum)

Maternal factor	Control	Obese
BW (g)	24.36 ± 0.31	34.23 ± 1.063*
Fasting glucose (mmol/l)	13.50 ± 0.63	15.17 ± 0.73
Kidney/BW (%)	0.73 ± 0.01	0.61 ± 0.02*
Liver/BW (%)	6.04 ± 0.37	6.52 ± 0.26*
Retroperitoneal fat/BW (%)	0.20 ± 0.02	1.29 ± 0.12*
Extrauterine fat/BW (%)	1.36 ± 0.11	5.60 ± 0.52*

Abbreviation: BW, body weight. *P<0.0001 compared with control. Results are expressed as mean ± S.E.M., *n*=28-30. Control: chow fed and Obese: HFD fed from 6 weeks prior to mating, throughout pregnancy and lactation.

Adipose tissue produces adipocytokines including adiponectin and leptin which have autocrine, paracrine, and endocrine effects and influence whole-body insulin sensitivity and hence the development of metabolic diseases [88]. Adiponectin promotes insulin sensitivity and has anti-inflammatory properties; decreased circulating levels are associated with obesity, insulin resistance, and T2D [88]. Pregnant obese dams have lower adiponectin levels and similarly offspring of obese mothers also have lower adiponectin levels [89]. In contrast, leptin plays important roles in modulating satiety and energy homeostasis. Although leptin is elevated in offspring of obese mothers, the offspring do not demonstrate reduced food intake suggesting that maternal obesity induces leptin resistance [36]. The mechanism of leptin resistance due to maternal obesity may be permanently programmed by intrauterine overnutrition as a result of alterations in neural circuitry that is similar to that induced by HFD consumption [90].

Adipocytes secrete inflammatory mediators including chemokines and cytokines which lead to both local and systemic inflammation [91]. Excessive lipids that cannot be stored in adipocytes are released into the blood and ectopically deposited in the liver, muscle, and pancreas. At these sites, inflammatory cytokines secreted by adipocytes, cause cellular functional injury. Together these metabolic abnormalities lead to insulin resistance and the predisposition toward metabolic disorders, which may lead to end-organ effects such as cardiovascular disease and CKD [92].

### The placenta and programming by maternal obesity

The placenta is the gatekeeper between the maternal and fetal circulation. It modulates the delivery of oxygen and nutrients including glucose, amino acids, free fatty acids, and hormones such as insulin and glucagon, and glucocorticoids from the maternal circulation to the growing fetus. In exchange, the placenta is responsible for transferring carbon dioxide, urea, waste products, and hormones from the fetal circulation to the maternal circulation for clearance. The placenta is now recognized as an integral programming agent for chronic disease in offspring and in particular, placental efficiency is a predictor of disease. Maternal obesity is known to modulate how the placenta forms and functions [93].

In general, placental function relates to how it delivers oxygen and nutrients to the fetus. Placental transport is dependent on a number of factors including placental size and function, maternal nutrient availability, and the stage of gestation [94]. Placental transport of key nutrients is not as simple as concentration gradients from maternal to fetal circulation, although for nutrients such as glucose there is a strong relationship between maternal and fetal glucose levels [95]. Most relevant to the setting of maternal obesity, is the transport of glucose and fats across the placenta.

Diffusion of glucose across the placenta takes place readily and results in increased fetal growth. The insulin-independent glucose transporter (GLUT) 1 (GLUT1) has been identified as the main transporter for glucose in the placenta [95]. In GDM, it has been demonstrated that GLUT1 is up-regulated probably as a result of hormonally driven mechanisms such as increased insulin and insulin-like growth factor-1 (IGF-1). In obese mothers without GDM, GLUT1 expression correlated with birth weight [96]. However, the placental role in transferring glucose from mother to fetus is yet more complex where fetal hyperinsulinemia, in response to fetal hyperglycemia can steepen the glucose gradient, known as glucose steal [97]. This is because hyperinsulinemia in the fetus is likely to accelerate glucose clearance into fetal tissues by increasing fat accumulation predominantly in adipose tissue and in the liver, which will consequently increase the fetal glucose steal. It has been postulated that even in the presence of normal maternal glucose levels, fetal hyperinsulinemia will still lower fetal glucose concentrations, thus a high-glucose gradient and an exaggerated glucose steal are sustained [97]. Furthermore, in the setting of a large maternal–fetal glucose gradient, maternal postprandial glucose peaks may even be blunted further exacerbating the fetal exposure to glucose though masking the phenomenon in the mother.

The placenta is highly permeable to free fatty acids, transfer of which is gradient dependent. In general, the pregnant state is associated with mobilization of lipids, cholesterol, and free fatty acids into the maternal circulation [98]. Lipoprotein metabolism is up-regulated. Maternal lipoproteins do not pass directly across the placenta, rather lipoprotein receptors, lipases, and fatty acid-binding transport proteins within the placenta allow placental uptake of triglycerides and cholesterol and passage to the fetus. In fact, the placenta also has the ability to re-esterify and store lipids for later fetal use [99]. Maternal obesity is associated with even greater increases in maternal lipid mobilization and triglyceride. Low-density lipoprotein (LDL) and free fatty acids are all increased in obese compared with normal weight pregnant women [98]. In animal models as well as humans, maternal obesity increases the expression of free fatty acid transporters within the placenta, including CD36, fatty acid transport proteins 1 and 4, which are associated with increased circulating lipids in fetal serum [100–104].

## Cellular mechanisms of developmental programming affected by maternal obesity

### Inflammation

Obesity is associated with chronic low-grade inflammation largely mediated by excess adipose tissue [105]. Increased inflammatory markers are evident in the placentae of obese mothers [93,106,107]. Enhanced placental expression of pro-inflammatory cytokines, and expression of a critical signaling molecule in the inflammatory pathway, Toll-like receptor 4, alongside increased macrophage accumulation has been demonstrated in obese HFD-fed non-human primates [108,109]. Furthermore, cord blood levels of inflammatory markers also are reported to be altered by exposure to an obese mother [110,111]. Dosch et al. [111] found that cord serum levels of key inflammatory cytokines were increased in neonates of obese mothers at cesarean section (BMI >35 at the time of delivery). The mechanisms by which chronic low-grade inflammation influences placental function and how inflammatory mediators are transmitted to offspring and perpetuated to increase chronic disease risk later in life are poorly understood. Of particular interest, is the emerging evidence that maternal obesity may lead to alterations in the maternal DNA methylome which thereby influences placental gene [112].

### Oxidative stress

An imbalance between reactive oxygen species and antioxidant defense mechanisms leads to cellular damage via oxidative stress. Markers of oxidative stress have been shown to be increased in the placentae of obese mothers [113]. In a study of overweight and obese Spanish women, reduced placental expression of mammalian target of rapamycin and up-regulation of sirtuin 1 and uncoupled protein 2 were demonstrated. The implication of their altered regulation suggests increased placental oxidative stress, given the known role of these genes in up-regulating cellular antioxidant defense mechanisms [114]. Interestingly, in this study they did not find increased inflammatory cytokines in the cord blood of the neonates exposed to maternal obesity.

Mitochondria are intracellular organelles extensively involved in cellular metabolism and oxidative stress defense as a result of inevitable free radical leakage in the process of cellular respiration. Mitochondrial dysfunction has been demonstrated as early as embryogenesis by maternal obesity [115]. Furthermore, the placentae of obese mothers have higher levels of oxidative stress and impaired mitochondrial respiration in both rodent and human placenta [116–118]. The outcome of increased oxidative stress and dysfunctional repair mechanisms is likely to be impaired placental function, which may thereby lead to unhealthy fetal growth and development.

### Changes in epigenetic regulation

Epigenetics is the study of heritable changes in gene expression that are not due to changes in the DNA sequence [119]. Epigenetic modification can occur via DNA methylation, histone modification, or by the influence of miRNA and small non-coding regions of the genome previously regarded as ‘junk’ though now recognized as important regulators of gene expression itself. DNA methylation typically occurs at CpG dinucleotide sites (regions of DNA where a cytosine nucleotide is followed by a guanine nucleotide) via the action of DNA methyltransferase enzymes. DNA methylation is usually associated with down-regulation or silencing of gene expression via direct methylation at non-CpG sites. Histone modification results in altered compaction of DNA around histones, preventing or enabling gene activation. Changes to histone structure as a result of various processes including acetylation, methylation, ubiquitylation, and phosphorylation lead to chromatin and nucleosome restructure which influences binding of transcription factors which can have numerous effects on gene regulation [119]. There is a complex interplay between epigenetic processes such that coupling between DNA methylation and histone modification increases the complexity of gene regulation. With respect to fetal programming, epigenetic changes are increasingly demonstrated to play an influential role in modulating gene expression which may persist from early life *in utero* through to adulthood.

In human studies, maternal nutrition as early as conception, can modulate DNA methylation of important genes. In a series of elegantly designed experiments taking place in a remote community in the Gambia, maternal nutritional status at the time of conception was shown to alter methylation status of a host of genes in the cord blood of offspring [120–122]. In rural communities in the Gambia, the combination of self-sufficient food source (without external influence) and reliance on stored foods during the rainy season lead to profound annual variations in the intakes of macro- and micronutrients. First, it was established that the season of conception (reflecting variation in the dietary intake and nutritional status of women at the time of conception) significantly influenced the methylation status in multiple genes in children [122]. Second, a randomized, controlled trial where Gambian women were given micronutrient supplementation, determined that particular candidate genes had altered methylation status as a result of the supplement as measured in cord blood of offspring [120]. Thereafter, a large randomized, controlled trial carried out in the Gambian communities demonstrated the powerful effect of periconceptional maternal nutrition on DNA methylation in offspring blood and hair and was predicted by periconceptional maternal plasma concentrations of key micronutrients, such as homocysteine, folate, and B vitamins [120].

A very powerful example of the potential effects of epigenetic modification comes from animal experiments involving the Agouti mouse. The wild-type *Agouti* gene encodes a paracrine-signaling molecule that produces either black or yellow fur [123]. When the *agouti* gene is in its normal methylated state, the coat is brown and the mouse has low risk of metabolic disease. In contrast, if the *agouti* gene is unmethylated, the mouse is yellow-furred and obese with dysregulated metabolism. When pregnant yellow-furred Agouti mice are fed methyl-rich diet, they produce mostly healthy brown-furred offspring. However, if fed methyl-poor diet the offspring are yellow furred and are at increased risk of obesity. In the human studies conducted in the Gambia, interestingly higher maternal BMI was significantly associated with hypomethylation status in the serum of the offspring [121]. To date, it is unclear if epigenetic changes established *in utero* are persistent throughout life or can be modulated by postnatal environmental factors.

## Clinical implications

**Figure 2:**
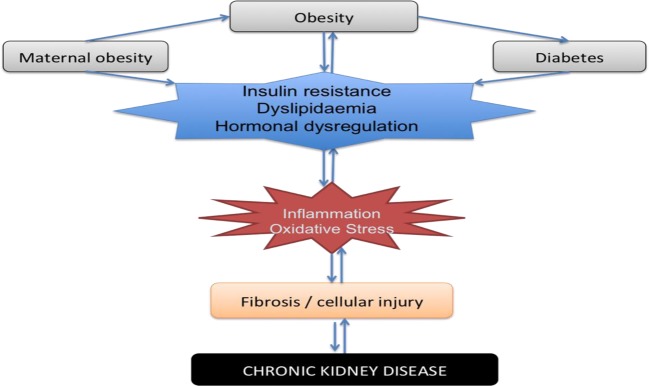
Influencing factors and cellular mechanisms leading to the development of CKD in offspring

There is firm evidence that maternal obesity influences the development of chronic disease in offspring, and our animal models of maternal obesity have now extended this knowledge to include developmental programming of renal disease. There is considerable evidence related to the pathways of inflammation, oxidative stress, and fibrosis with regard to the pathogenesis of CKD [124,125]. Maternal obesity is an important modulator of these pathways and hence should be considered a contributor to future CKD risk in the offspring (see Figure 2). Further studies are needed to explore the critical components of *in utero* exposure underlying the influence of maternal obesity on renal outcomes, specifically determining the role of hypertension in CKD risk in offspring. The role of epigenetics in modulating the effects of maternal obesity on metabolic disease in offspring is an important area for future research and no doubt will provide explanation for the transgenerational propagation of metabolic disease.

As all-important gatekeeper between the maternal and fetal circulations, the placenta is likely to play a critical role in fetal programming as previously described. An objective for future studies is to determine the role of the placenta in orchestrating the effect of perturbations related to maternal obesity on metabolic programming within the fetal kidney. There are several unanswered questions related to this concept. Are all periods of gestation as important for the deleterious renal effects of maternal obesity? For example, is early gestation most important when placental implantation is occurring or is late gestation more important when final kidney maturation is taking place? Another question arises: if weight loss occurs prior to conception, will this resolve the negative programming effects of maternal obesity on the renal health of offspring? There is low level of evidence to suggest that this may not be the case and invokes the concept of irreversible epigenetic modifications with the potential to lead to transgenerational propagation of obesity and renal disease [126,127]. Transmission of epigenetic modifications from mother to child may shift the population phenotype, particularly if occurring in the mtDNA.

Specific targets to reduce inflammation and oxidative stress are needed to prevent the harmful effects of maternal obesity on renal health, and other deleterious metabolic effects. Agents safe in pregnancy that can reduce inflammation, oxidative stress, or reverse adverse epigenetic modification, may be useful to prevent developmental programming of maternal obesity impacting on renal health in offspring. Finally, its far-reaching consequences for disease propagation to subsequent generations calls for unity of industry, academia, and public health to come together with policymakers and governments to devise public health strategies to reduce obesity, particularly in women of reproductive age.

## Abbreviations

BMI: body mass index
CKD: chronic kidney disease
GDM: gestational diabetes mellitus
GLUT: glucose transporter
HFD: high-fat diet
NAFLD: non-alcoholic fatty liver disease
PPARγ: proliferator-activated receptor-γ
T2D: type 2 diabetes
